# Estimated Influenza Illnesses and Hospitalizations Averted by Vaccination — United States, 2013–14 Influenza Season

**Published:** 2014-12-12

**Authors:** Carrie Reed, Inkyu Kevin Kim, James A. Singleton, Sandra S Chaves, Brendan Flannery, Lyn Finelli, Alicia Fry, Erin Burns, Paul Gargiullo, Daniel Jernigan, Nancy Cox, Joseph Bresee

**Affiliations:** 1 Influenza Division, National Center for Immunization and Respiratory Diseases, CDC; 2 Immunizations Services Division, National Center for Immunization and Respiratory Diseases, CDC

The Advisory Committee on Immunization Practices recommends annual influenza vaccination for all persons aged ≥6 months to reduce morbidity and mortality caused by influenza in the United States (1). CDC previously developed a model to estimate that annual influenza vaccination resulted in 1.1–6.6 million fewer cases and 7,700–79,000 fewer hospitalizations per season during the 2005–2013 influenza seasons (2,3). For the 2013–14 influenza season, using updated estimates of vaccination coverage, vaccine effectiveness, and influenza hospitalizations, CDC estimates that influenza vaccination prevented approximately 7.2 million illnesses, 3.1 million medically attended illnesses, and 90,000 hospitalizations associated with influenza. Similar to prior seasons, fewer than half of persons aged ≥6 months are estimated to have been vaccinated.* If influenza vaccination levels had reached the *Healthy People 2020* target of 70%, an estimated additional 5.9 million illnesses, 2.3 million medically attended illnesses, and 42,000 hospitalizations associated with influenza might have been averted. For the nation to more fully benefit from influenza vaccines, more effort is needed to reach the *Healthy People 2020* target.

The methods used have been described in detail previously (2) and are outlined briefly in this report for the 2013–14 season. First, CDC estimated the number of illnesses, medically attended illnesses, and hospitalizations associated with influenza that occurred in the United States during the 2013–14 influenza season. Laboratory-confirmed influenza-associated hospitalization rates by age group were obtained from FluSurv-NET, a collaboration between CDC, the Emerging Infections Program Network, and selected health departments in 13 geographically distributed areas in the United States that conduct population-based surveillance.† Hospitalization rates were adjusted for underreporting based on the frequency and sensitivity of influenza testing in surveillance hospitals during two post-pandemic seasons (4); hospitalization rates were multiplied by a factor of 2.1 for ages <20 years, 3.2 for 20–64 years, and 5.3 for ≥65 years. In previous years, influenza hospitalization rates were multiplied by a factor of 2.7 based on data collected during the 2009 influenza pandemic that were not age-specific (2,3). Data were collected during two post-pandemic seasons to update these multipliers (4) because influenza testing might not be as common as during the pandemic and the previous multipliers might have underestimated hospitalizations in nonpandemic years. The updated multipliers were similar to the previous estimates for children and younger adults, but indicate that estimated hospitalization rates among older adults in recent seasons were too low.

Adjusted rates were applied to the U.S. population by age group to calculate numbers of hospitalizations. The numbers of influenza illnesses were estimated from hospitalizations based on previously measured multipliers that reflect the estimated number of ill persons per hospitalization in each age group: 143.4 for 0–4 years; 364.7 for 5–19 years; 148.2 for 20–64 years, and 11.0 for ≥65 years (2). The numbers of persons seeking medical care for influenza were then calculated using age group-specific data on the percentages of persons with a respiratory illness who sought medical attention, which were estimated from results of the 2010 Behavioral Risk Factor Surveillance Survey: 67% for ages 0–4 years; 51% for ages 5–19 years; 37% for ages 20–64 years, and 56% for ages ≥65 years (2).

Second, 2013–14 estimates of vaccination coverage through April 2014 and end-of-season vaccine effectiveness data were used to estimate how many persons were not protected by vaccination during the season and thus were at risk for influenza illness, medically attended illness, and influenza-related hospitalization. The rate of each outcome among persons at risk was then used to estimate the number of influenza-associated outcomes that would have been expected in the same population if no one had been protected by vaccination. Estimates of 2013–14 influenza vaccination coverage were based on self-report or parental report of vaccination status using data from the National Immunization Survey for children aged 6 months–17 years and Behavioral Risk Factor Surveillance Survey data for adults aged ≥18 years, and varied from 37% to 70%, depending on the age group (Table 1) (5). Vaccine effectiveness estimates for the 2013–14 season were derived from the U.S. Influenza Vaccine Effectiveness Network, a group of five academic institutions that conduct annual vaccine effectiveness studies (5,6). The network estimates the effectiveness of vaccination for preventing real-time reverse transcription polymerase chain reaction–positive influenza among persons with acute respiratory illness of ≤7 days duration seen in outpatient clinics in communities in five states. Vaccine effectiveness estimates were updated to include data collected through the end of season and ranged from 39% (95% confidence interval [CI] = −6%–65%) for persons aged ≥65 years to 56% (CI = 37%–69%) for persons aged 5–19 years (Influenza Division, National Center for Immunization and Respiratory Diseases, CDC; unpublished data; 2014).

Finally, the averted outcomes attributable to vaccination were calculated as the difference between outcomes in the hypothetical unvaccinated population and the observed vaccinated population. Calculations were stratified by month of the year to account for annual variations in the timing of disease and vaccination and then summed across the whole season. The prevented fraction was calculated as the number of averted illnesses divided by the total illnesses that would have been expected in an unvaccinated population.

During October 2013–May 2014, influenza vaccination resulted in an estimated 7.2 million (CI = 5.1–9.9) fewer illnesses, 3.1 million (CI = 2.1–4.4) fewer medically attended illnesses, and 90,068 (CI = 51,231–144,571) fewer hospitalizations (Table 2) associated with influenza. Overall, 16.9% (CI = 15.3%–18.0%) of these adverse health outcomes associated with influenza were prevented. Using the same model, if vaccination levels had instead reached the *Healthy People 2020* target of 70%,§ an additional 5.9 million illnesses, 2.3 million medically attended illnesses, and 42,000 hospitalizations might have been averted.

Although 17% of the averted illnesses and 24% of averted medically attended illnesses were among children aged 6 months–4 years and persons aged ≥65 years (two groups known to be at higher risk for complications), these two age groups accounted for 60% of averted hospitalizations. Persons aged ≥65 years accounted for 55% of all hospitalizations prevented.

## Discussion

During the 2013–14 season, influenza activity peaked in late December, and the influenza A (H1N1)pdm09 virus predominated in the United States for the first time since the 2009 pandemic (7). There were somewhat fewer estimated influenza-associated hospitalizations overall than during the previous season (3), which had been a moderately severe season during which influenza A (H3N2) viruses predominated. In 2013–14, however, rates of hospitalization for adults aged 20–64 years were 1.3–5.5 times higher than during previous reported seasons (2,3). In addition, 109 influenza-associated pediatric deaths (deaths among persons aged <18 years) were reported to CDC (7), most of which were associated with influenza A (H1N1)pdm09.

During the 2013–14 season, a 17% overall reduction in illnesses resulted in a large number of prevented influenza-associated medical visits and hospitalizations. The prevented fraction was similar to recent seasons (2,3) and was highest among children aged <5 years (25%) and lowest for adults aged 20–64 years (15%). Fewer than half of adults aged 20–64 in the United States are vaccinated each season despite a recommendation for universal influenza vaccination for persons aged ≥6 months (1). Adults aged 20–64 years make up approximately 60% of the U.S. population and during the 2013–14 season accounted for 77% of estimated influenza illnesses and 46% of hospitalizations (Table 1). This sizeable population has the lowest influenza vaccination coverage (37%) and therefore the most potential gains through use of strategies known to improve coverage. Such strategies include ensuring that all those who visit a health care provider during the influenza season receive an influenza vaccination recommendation from their provider, using patient reminder/recall systems, using immunization information systems, and expanding access through use of nontraditional settings for vaccination (e.g., pharmacies, workplaces, and schools) to reach persons who might not visit a physician’s office during the influenza season.¶

What is already known on this topic?

Influenza vaccination has been a central tool for influenza prevention in the United States for more than 50 years. Previously, CDC estimated that annual influenza vaccination resulted in 1.1–6.6 million fewer cases and 7,700–79,000 fewer hospitalizations annually during the 2005–2013 influenza seasons.

What is added by this report?

Using surveillance data, vaccination coverage survey data, and vaccine effectiveness estimates collected during the 2013–14 season, estimates of the impact of influenza vaccination for the 2013–14 season were generated. Vaccination during the 2013–14 season resulted in an estimated 7.2 million fewer cases of influenza, 90,000 fewer hospitalizations, and 3.1 million fewer medically attended cases than would have been expected without vaccination. If vaccination levels had reached the *Healthy People 2020* target of 70%, an additional 5.9 million illnesses, 2.3 million medically attended illnesses, and 42,000 hospitalizations might have been averted.

What are the implications for public health practice?

Although influenza vaccination prevented millions of illnesses and tens of thousands of hospitalizations in 2013–14, there is a need for increased vaccination coverage and more effective vaccines to further reduce the burden of influenza.

During 2013–14, the vaccine effectiveness point estimate was lowest among persons aged ≥65 years (39% [CI = −6%–65%]). Almost half of the 2013–14 estimated hospitalizations (Table 1), and in many years >90% of influenza deaths (9), occur among adults aged ≥65 years. A recent study using this same analytic framework showed that even with very low vaccine effectiveness among older adults (10%), current influenza vaccination coverage in older adults can still help to prevent a sizeable number of influenza hospitalizations during moderately severe seasons (8). Vaccination coverage rates are relatively high in this vulnerable population; therefore, major gains in preventing severe outcomes in this age group will require vaccines with better efficacy for persons in this age group.

The findings in this report are subject to at least five limitations. First, influenza vaccination coverage estimates were derived from reports by survey respondents, not vaccination records, and are subject to recall bias. Furthermore, these estimates are based on telephone surveys with relatively low response rates; although weighting adjustments were designed to improve representativeness of the sample, they might not completely eliminate nonresponse bias. Estimates of the number of persons vaccinated based on these survey data have exceeded the actual number of doses distributed, indicating that coverage estimates might be somewhat lower than those used in this report and might overestimate the numbers of illnesses and hospitalizations averted by vaccination. Second, this model only calculates outcomes directly averted among persons who were vaccinated. If there is indirect protection from decreased exposure of unvaccinated persons to infectious persons in a partially vaccinated population (i.e., herd immunity), the model would underestimate the number of illnesses and hospitalizations prevented by vaccination. Third, vaccine effectiveness was lower for adults aged ≥65 years; the effectiveness might continue to decrease with age, reaching very low levels among the oldest adults with the highest rates of influenza vaccination; thus, the model might have overestimated the effect in this group. Fourth, this model assumes that vaccine effectiveness is the same for all outcomes. Finally, the fraction of persons with influenza who seek medical care was estimated from data collected during the 2009 pandemic, although the values were similar to those derived from surveys conducted the following season (10). If health care seeking differed during the 2013–14 influenza season, the number of influenza medical visits in the population might have been overestimated or underestimated.

Influenza vaccination prevented a substantial amount of influenza disease in the United States last season, including an estimated 3 million medical visits and 90,000 hospitalizations. Although vaccines with increased effectiveness are needed, much can be done to maximize influenza prevention during the upcoming 2014–15 season. In particular, efforts to increase vaccination coverage will further reduce the burden of influenza, especially among adults aged 20–64 years, who continue to have the lowest influenza vaccination coverage. Although the timing and intensity of influenza virus circulation for the 2014–15 season cannot be predicted, peak weeks of influenza activity have occurred in January through March in >75% of seasons during the past 30 years, and significant circulation can occur as late as May. Therefore, vaccination should continue to be offered through the peak periods of influenza virus circulation and as long as influenza viruses are reported to be circulating for the current season.

## Figures and Tables

**TABLE 1:** Variables affecting impact of influenza vaccination, by age group — United States, 2013–14 influenza season

Age group	Vaccination coverage*		Vaccine effectiveness†		Total population§	Hospitalization rate (per 100,000)¶	Estimated hospitalizations		Estimated medically attended cases**		Estimated cases††	
				
	%	(95% CI)	%	(95% CI)			No.	(95% CI)	No.	(95% CI)	No.	(95% CI)
6 mos–4 yrs	70.1	(68.8–71.4)	47	(14–67)	17,762,071	77.8	13,811	(10,589–17,853)	1,327,271	(1,014,859–1,722,629)	1,981,002	(1,518,891–2,560,814)
5–19 yrs	51.2	(50.4–52.0)	56	(37–69)	62,379,999	18.1	11,310	(8,559–14,765)	2,103,728	(1,587,897–2,759,290)	4,124,957	(3,121,675–5,384,883)
20–64 yrs	36.8	(36.4–37.2)	52	(42–61)	189,176,678	96.9	183,320	(145,397–234,061)	10,052,182	(7,883,113–12,806,943)	27,168,060	(21,547,842–34,687,824)
≥65 yrs	64.7	(64.1–65.3)	39	(0–65)	44,704,074	422.3	188,795	(147,987–259,760)	1,162,978	(899,730–1,615,888)	2,076,747	(1,627,860–2,857,358)
**All ages**					**314,022,822**	**126.5**	**397,236**	**(312,532–526,439)**	**14,646,159**	**(11,385,599–18,904,750)**	**35,350,766**	**(27,816,268–47,490,879)**

**Abbreviation:** CI = confidence interval.

* Season-cumulative vaccination coverage rates calculated using data from the National Immunization Survey for children aged 6 months–17 years and from the Behavioral Risk Factor Surveillance System for adults aged ≥18 years. Model uses incremental monthly age-specific values. Estimates of the cumulative monthly proportion vaccinated through end of April of each season were developed using the Kaplan-Meier product limit method for receipt of most recent reported influenza vaccination. Negative lower CIs were revised to 0.

† Based on methods for estimating vaccine effectiveness in the United States by age group. Values for the 2013–14 season are updated with data through the end of the season. Negative lower CIs were revised to 0.

§ Calculated from U.S. Census Bureau annual estimates of the resident population by single year of age and sex for April 1, 2010–July 1, 2013, available at http://factfinder2.census.gov/faces/tableservices/jsf/pages/productview.xhtml?pid=PEP_2013_PEPSYASEXN&prodType=table.

¶ Season-cumulative hospitalization rates calculated using data from the CDC Influenza Hospitalization Surveillance Network (FluSurv-NET) and adjusted for underreporting. The underreporting adjustment multiplier was calculated during two post-pandemic seasons: 6 mos–19 yrs: 2.1 (95% CI = 1.8–2.6), 20–64 yrs: 3.2 (95% CI = 2.5–4.3), and ≥65 yrs: 5.3 (95% CI = 4.1–7.8). Model uses month-specific and age-specific values.

** Based on the percentage of persons with an influenza-like illness who reported seeking medical care as reported through the Behavioral Risk Factor Surveillance System.

†† Based on the estimated number of hospitalizations and age-specific case-hospitalization ratios: 143.4 for 0–4 years, 364.7 for 5–19 years, 148.2 for 20–64 years, and 11.0 for adults ≥65 (2,3).

**TABLE 2:** Estimated number and fraction of influenza cases averted by vaccination — United States, 2013–14 influenza season

Age group	Averted cases		Averted medically attended cases		Averted hospitalizations		Fraction prevented	
			
	No.	(95% CI)	No.	(95% CI)	No.	(95% CI)	%	(95% CI)
6 mos–4 yrs	657,701	(366,554–1,013,644)	440,660	(245,692–680,502)	4,585	(2,555–7,067)	24.9	(18.5–29.3)
5–19 yrs	1,185,034	(837,466–1,638,601)	604,368	(423,423–841,847)	3,249	(2,296–4,493)	22.3	(20.9–23.6)
20–64 yrs	4,786,265	(3,626,912–6,259,499)	1,770,918	(1,331,958–2,330,947)	32,296	(24,473–42,237)	15.0	(14.4–15.3)
≥65 yrs	549,317	(240,964–998,517)	307,618	(134,114–561,318)	49,938	(21,906–90,774)	20.9	(12.1–27.0)
**All ages**	**7,178,318**	**(5,071,896–9,910,260)**	**3,123,563**	**(2,135,186–4,414,614)**	**90,068**	**(51,231–144,571)**	**16.9**	**(15.3–18.0)**

**Abbreviation:** CI = confidence interval.

## Footnotes

* Annual estimates of influenza vaccination coverage in the United States can be viewed at http://www.cdc.gov/flu/fluvaxview/coverage-1314estimates.htm. Methods for estimating season-specific influenza vaccination coverage and descriptions of National Immunization Survey and Behavioral Risk Factor Surveillance Survey data are available at http://www.cdc.gov/mmwr/pdf/ss/ss6204.pdf.

† National, regional, and state influenza surveillance data are available at http://www.cdc.gov/flu/weekly/fluactivitysurv.htm.

§ *Healthy People 2020* objectives for increasing the percentage of children and adults vaccinated annually against seasonal influenza (IID-12.11 and IID-12.12) are available at http://www.healthypeople.gov/2020/topics-objectives/topic/immunization-and-infectious-diseases/objectives.

¶ Evidence-based strategies for improving vaccination coverage are described in the *Community Guide for Preventive Services*, available at http://www.thecommunityguide.org/index.html.
